# Treatment of Sprains*Abridged from a paper read before the Lancashire and Cheshire branch.

**Published:** 1880-12

**Authors:** R. Dacre Fox

**Affiliations:** Edinburgh. Surgeon to the Manchester Southern Hospital; Chief Medical Officer to the Manchester, Sheffield and Lincolnshire Railway Company, etc.


TREATMENT OF SPRAINS.* By R. DACRE FOX, F.R.C.S., Edinburgh. Surgeon to the Manchester Southern Hospital; Chief Medical Officer to the Manchester, Sheffield and Lincolnshire Railway Company, etc.

The frequency with which sprains occur in general practice, and the somewhat unsatisfactory results of the treatment ordinarily adopted, induce me to bring forward a method that I have used in a great many cases with considerable success. Sprains may be broadly divided into two kinds, mild and severe; the former consisting merely of a temporary overdistension of the parts around a joint, which rest and anodyne applications usually soon cure; the latter involving, as I believe, much more serious pathological results, which the following plan is specially contrived to obviate.

The effects of a severe sprain are, that the fibrous ligaments controlling the movements of the joint and binding the tendons in their grooves become overstretched, swollen and softened; the cellular tissue about the ligaments and in the tendon-grooves becomes œdematous; and plastic material is exuded; while, as a consequence of these changes, the tendons are displaced in their beds. If this condition be not actively treated, it may, and often does, lead to continued lameness, due, in all probability, partly to a diminution in the caliber of the tendon-groove, with impaired muscular action, and partly to the torn ligaments and bruised cellular tissue having undergone changes which render them incapable of adapting themselves to the movements of the

* Abridged from a paper read before the Lancashire and Cheshire branch.

joint, which are consequently impeded. I believe this result may be prevented by the application of firm direct equal pressure, applied manually at first, and kept up and controlled by pads placed in the line of the tendons, and kept in position by properly shaped plasters and bandages, and sometimes by splints. This pressure helps to disperse the œdema, to replace the tendon in its normal position, to hasten the absorption of any plastic exudation, and thus to prevent diminution in the caliber of the tendon-groove. I cannot say this is a novel method of treatment; but I think it is one not usually practiced, partly because it entails the expenditure of much time and trouble, and partly, I feel sure, because there is and has been a tendency to underestimate the inconvenience and distress arising from a badly sprained joint.

The common practice, in treating a sprain, is to put on a bandage, telling the patient to take it off if the joint becomes painful, and to substitute warm water fomentations. When the swelling has subsided, if the injury be not so slight as to be already cured, a liniment or the application of iodine is generally ordered. Very frequently the tight bandage causes inflammation, while the rubbing and painting are practically useless. There are numbers of cases of slight sprain, indeed, which will get well with comparatively little treatment or none at all; but in that more severe form where, after an inflammatory or at least exceedingly hyperæmic stage, swelling takes place, with the results I have described, the application of these remedies does not prevent the joint from being left rigid, painful and unfit for use for a very long period. Now it is, as I have said, in preventing all this, that the plan of treatment by direct, equal and continuous pressure will be found exceedingly valuable; for, where it has been properly carried out, I have always found that the joint returns quickly to its normal condition—pain being speedily relieved, and rigidly prevented. The treatment may be divided into two stages; the first lasting from a day to a week or longer, during which the treatment has to be directed to averting inflammation by rest, warm applications, anodyne lotions, etc.; the second commencing when the joint has become cold, swollen and painful on movement—in fact, when the injury has assumed a more or less chronic character. It is during this second period that I believe the active treatment I advocate ought to be employed. It is important not to commence this until the surface-heat is normal; for undoubtedly, when any tendency to inflammation exists in the tendon-sheath, pressure aggravates it, and I have known it to lead to untoward results.

It is of course impossible, within the limits of this paper, to describe the special adaptation of this method to each joint; but I will take as an illustration the ankle. If a wire be passed round the joint so as to impinge on the two malleoli and the tendo Achillis, it will define three or four well-marked hollows: one on each side of the tendo Achillis behind each malleolus, one in front of the fibula, with a fourth shallower one in front of the tibia. When the ankle is severely sprained these fossæ become obliterated, and are filled up with effusion, overstretched ligaments and displaced tendons.

Observation has led me to believe that there are very few sprained ankles in which muscular displacement to some degree does not take place. It most commonly occurs in front of the outer malleolus, involving the outer part of the annular ligament, the extensor longus digitorum, and the anterior fasciculus of the external lateral ligament; next, perhaps the posterior peroneo-tarsal ligament and structures behind the external malleolus. Cases of similar overstretching and displacement on the inner side of the ankle are happily rare; but in gravity they bear much the same relation to the former as a Pott's dislocation does to a simple fractured fibula. I will assume an ankle-joint has sustained a severe sprain all round, and has arrived at the chronic stage; modifications of the treatment of such a case will meet all that are likely to occur. To carry out the first principles of treatment by direct, equal and continuous pressure, it is clear the fossæ mentioned above must be filled, or rather their sites covered by pads so as to cause the retaining plasters, bandages and splints to exercise equal pressure everywhere. By making pressure with the thumb from below upwards in the line of the fossæ, a good deal of the œdema may be squeezed away and the displaced tendons in some degree restored. I make, as a rule, five pads (of tow and lint or leather): two about four inches long by one inch wide (one a little shorter than the other, so as to be better adapted to the curve extending upward from the dorsum of the foot to the crest of the tibia); another shorter, broader and thinner, to place over the tibialis anticus and extensor proprius pollicis; and two, three or four inches long and bolster-shaped, to fill in the posterior fossæ on each side of the tendo Achillis. It is often advisable, in old-standing cases, to supplement the pads by strips of plaster to ensure firmer pressure. Both pads and strips of plaster should be made exactly to fit, as, if too large, they are useless, from the pressure being too diffused; and, if too small, they exercise too little pressure. A moment's consideration will render this obvious. If too large a pad, for instance, be placed over the outer postmalleolar fossa, its edges rest on the tendo Achillis and outer malleolus like the piers of an arch, leaving the fossa itself untouched. To keep these pads in their place, I use a long extended half-moon-shaped piece of plaster (emplastrium saponis spread on leather), long enough for the ends to overlap in front when the heel is placed in the center, and a narrow oblong piece above this, placed round the lower part of the leg, to cover the upper part of the pads. The handiest way to apply the pads is to place an India-rubber band above the ankle, to slip the pads under it, and then, planting the heel in the center of the curved plaster, to bring the two ends across the front of the joint so as to overlap. The pads having been secured in position, the elastic ring is to be cut, and the oblong piece of plaster put on so as to encircle their upper ends; lastly the whole ankle is to be firmly bandaged. Amongst the working classes, or in the case of an uncontrollable patient, it is advisable to apply two thin splints over the anterior pads, keeping them in position by a long strip of adhesive plaster. Where there is much superficial ecchymosis, where there are bullæ or where there is unhealthy-looking skin, instead of using soap-plaster, the pads may be kept in position and pressure maintained by a piece of lint on which ointment has been spread. Calamine ointment made stiffly is clean, and not uncomfortably greasy. If, as occasionally happens, even this should cause irritation, warm wet lint covered by oiled silk may be advantageously used over the pads, and secured by a firm bandage; but neither of the applications can compare in efficiency with the soap plaster spread on leather.

It is, as I have said, impossible in the limits of this paper to describe the method of adaptation of the pads to all the different joints; but a very little consideration will suggest the shape, size and thickness necessary to be employed in each case.

